# In-Situ Growth of ZnO Whiskers on Ti_2_ZnC MAX Phases

**DOI:** 10.3390/ma16103610

**Published:** 2023-05-09

**Authors:** Yinan Ren, Zhihua Tian, Yan Zhang, Fushuo Wu, Hao Xie, Qianqian Zhang, Peigen Zhang, Zhengming Sun

**Affiliations:** 1 Jiangsu Key Laboratory of Advanced Metallic Materials, School of Materials Science and Engineering, Southeast University, Nanjing 211189, China; 2 Liyang Zichen New Material Technology Co., Ltd., Changzhou 213000, China

**Keywords:** Ti_2_ZnC MAX phase, crystal growth, in-situ growth, ZnO whiskers

## Abstract

ZnO whiskers have many applications, such as in medical and photocatalysis fields. In this study, an unconventional preparation approach is reported, realizing the in-situ growth of ZnO whiskers on Ti_2_ZnC. The weak bonding between the layer of Ti_6_C-octahedron and the Zn-atom layers leads to the easy extraction of Zn atoms from Ti_2_ZnC lattice points, resulting in the formation of ZnO whiskers on the Ti_2_ZnC surface. This is the first time that ZnO whiskers have been found to grow in-situ on Ti_2_ZnC substrate. Further, this phenomenon is amplified when the size of the Ti_2_ZnC grains is mechanically reduced by ball-milling, which bodes a promising route to prepare ZnO in-situ on a large scale. Additionally, this finding can also help us better understand the stability of Ti_2_ZnC and the whiskering mechanism of MAX phases.

## 1. Introduction

ZnO is a functional semiconductor with a wide band gap (3.37 eV) and a considerable exciton binding energy (60 meV) at room temperature [1,2], which can be used in sensors [3,4] and photocatalysis [5,6,7,8,9]. In addition, because of its nontoxicity and biocompatibility, ZnO can also be used in medical fields [10]. In 2006, Wang et al. [11] successfully used the tip of a conductive atomic force microscope probe to bend ZnO whiskers, perfectly converting this part of mechanical energy into electrical energy, and invented nanogenerators, which opened a door for the study of ZnO materials. ZnO whiskers have also been widely studied as an efficient photocatalyst for the degradation of organic pollutants in water treatment [5,6] by which organic pollutants are decomposed into small molecules and less-harmful products such as CO_2_ and H_2_O [12]. The preparation of ZnO is the foundation for all of its applications and may affect its application effect. Therefore, the preparation methods for ZnO are important and always draw attention from both academic and industrial circles.

The primary methods for preparing ZnO whiskers include vapor deposition [13,14,15] and the hydrothermal method [16,17,18]. Physical vapor deposition (PVD) consists of vaporizing the raw material of ZnO powder and then forming a ZnO nanomaterial from the gaseous state without a catalyst. Since ZnO has a high melting point (1975 °C), the temperature required for the synthesis of ZnO is very high [13]; as for chemical vapor deposition (CVD), in addition to the above physical changes, chemical reactions such as oxidation and compounding also occur. The formation and morphology of ZnO are affected by many factors such as temperature, gas pressure, gas flow, and catalyst [18]. Compared with vapor deposition, the hydrothermal method has the advantages of low temperature and simple equipment, and it is suitable for large-scale preparation. However, the hydrothermal process usually requires a seeded substrate for highly dense arrayed ZnO whiskers. The interface in the presence of seeds has defects, leading to low adhesion [19]. In addition, these methods may need to transfer the as-synthesized ZnO for some specific application purposes. Sometimes, it is significant to immobilize ZnO on substrates, for example when it is used as photocatalyst, to realize a sustainable operation as well as good photocatalyst recovery [20,21,22], because the recovery of photocatalysts after water treatment is complicated and costly [12,23]. Therefore, the in-situ preparation of ZnO is the requirement of the technology development.

Recently, an interesting phenomenon of whisker growth on MAX phases has drawn increasing attention [24,25,26,27,28]. The MAX phases are layered and hexagonal early transition-metal carbides and nitrides, with a general formula of M_n+1_AX_n_ (where n = 1, 2, 3; M: early transition metal; A: A-group element; X: C or N) [29,30,31,32]. They combine the excellent properties of metals and ceramics, such as good electrical conductivity, high temperature oxidation resistance, and corrosion resistance [33,34]. However, due to the weak bonding between the M_6_X-octahedron layer and the A-atom layer, A-site atoms can be easily released from the lattice points of the MAX phase during mechanical exfoliation [24,25,27]. So far, there have been some reports [26,27,28,35,36] confirming that A-site metal whiskers (such as Ga, Sn, and In whiskers) can grow on the corresponding MAX phases (Cr_2_GaC, Ti_2_SnC, and Ti_2_InC, respectively). Zn-containing MAX phases (e.g., Ti_2_ZnC, V_2_ZnC, and Ti_3_ZnC_2_) were successfully synthesized by Huang Qing et al. [37]. If Zn atoms can be extracted from the Ti_2_ZnC precursor and be made to oxidize in-situ, the in-situ preparation of ZnO whiskers on the MAX phase substrates may be realized. Considering that MAX phases are corrosion-resistant and conductive [29,33], they would be the ideal substrates for the specific applications of ZnO whiskers, and realizing ZnO whiskers grown in-situ on MAX phases would be beneficial for a host of applications that require the immobilization of ZnO whiskers.

Herein, Ti_2_ZnC was used to grow ZnO whiskers in-situ. The results bode a new method for the preparation of ZnO whiskers, which does not require very high temperature and gas flow like traditional vapor deposition and is expected to be promising in applications that require the immobilization of ZnO whiskers. In addition, the size of Ti_2_ZnC particles is reduced after ball-milling and more Zn atoms diffuse out of the Ti_2_ZnC, resulting in more ZnO whiskers being grown on the surface of the sample. The findings not only help us better understand the stability of Ti_2_ZnC but also help us further understand the whiskering mechanism of MAX phases, which would be utilized for ZnO whisker preparation.

## 2. Materials and Methods

Commercial powders (TiC: 99.9%, 300 mesh, Nanjing crystal chemical Co., Nanjing, China; Ti: 99.99%, ≥300 mesh, Aladdin, Shanghai, China; Al: 99.7%, 300 mesh, Zhongnuo new materials Co., Beijing, China) were used as raw materials to synthesize Ti_2_AlC following previous work [38,39]. First, the powders of TiC, Ti, and Al were mixed according to a molar ratio of 0.95:1.05:1.05. Afterwards, the mixture was placed in an alumina crucible and then sintered at 1400 °C for 1 h under the protection of Ar gas to obtain the precursor Ti_2_AlC. The as-synthesized Ti_2_AlC was mixed with ZnCl_2_ by a molar ratio of 1:1.5 using an agate mortar under the protection of nitrogen in a glovebox. The mixed Ti_2_AlC/ZnCl_2_ was treated at 550 °C for 5 h in an Ar atmosphere. Then, the reactant was washed with deionized water and dried at 40 °C, to exclude the interference of the elemental Zn in the as-synthesized Ti_2_ZnC, and an acid pickling treatment was carried out, soaking the as-synthesized Ti_2_ZnC in a 1 mol/L HCL solution for 5 h with continuous stirring. Finally, the pickled Ti_2_ZnC was collected by suction filtration and then dried in an oven under 40 °C, and thus the target MAX phase Ti_2_ZnC was obtained. The obtained Ti_2_ZnC powder was divided into two groups. One group of Ti_2_ZnC powder was directly cold-pressed into disc samples under 800 MPa and then heated at 400 °C for 48 h, and the other group was ball-milled and then pressed into disc specimens.

To reduce the grain size of the Ti_2_ZnC, it was ball-milled in stainless steel jars with steel milling balls, the ratio of the ball to the Ti_2_ZnC was 10:1, the rotating speed was 700 rpm, and the milling time was 18 h. After the ball-milling, the as-milled Ti_2_ZnC powder was cold-pressed into discs under 800 MPa and then heated at 400 °C for 48 h.

The phase composition of Ti_2_ZnC samples under different conditions was characterized by using an X-ray diffractometer (XRD, Haoyuan, DX–2700BH, Cu-K_α_ radiation) operating at 40 kV and 30 mA, wherein the 2-theta scanning range was 5° to 65°. The morphologies of samples were characterized by scanning electron microscopy (SEM, FEI Sirion 200, Hillsboro, OR, USA), wherein the applied electron beam acceleration voltage was 12–15 kV and the transmission electron microscope (TEM, Thermo Talos F200X, Waltham, MA, USA) equipped with an energy spectrum probe (EDS, Oxford X-Max 50, Abingdon, UK) was also used to further characterize ZnO whiskers.

## 3. Results and Discussion

**Figure 1:**
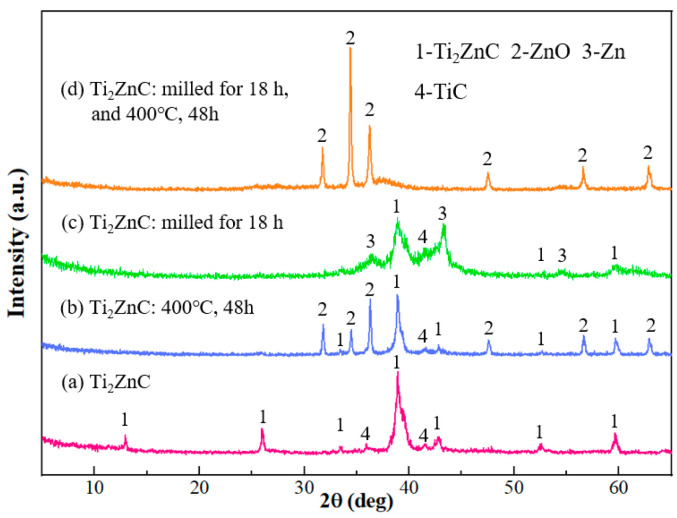
XRD patterns: (**a**) Ti_2_ZnC, (**b**) Ti_2_ZnC after aging for 48 h at 400 °C, (**c**) ball-milled Ti_2_ZnC (18 h), and (**d**) ball-milled Ti_2_ZnC (18 h) after aging for 48 h at 400 °C.

**Figure 2:**
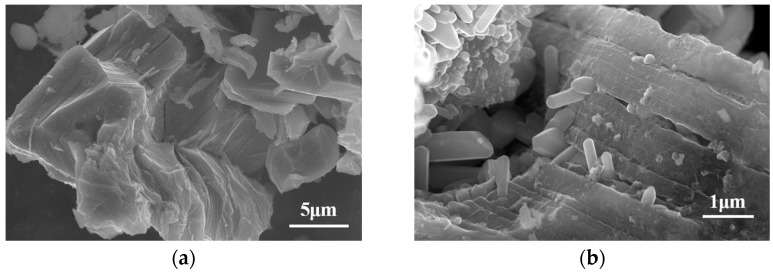
SEM characterization of (**a**) the Ti_2_ZnC and (**b**) the Ti_2_ZnC after being heated at 400 °C for 48 h.

**Figure 3:**
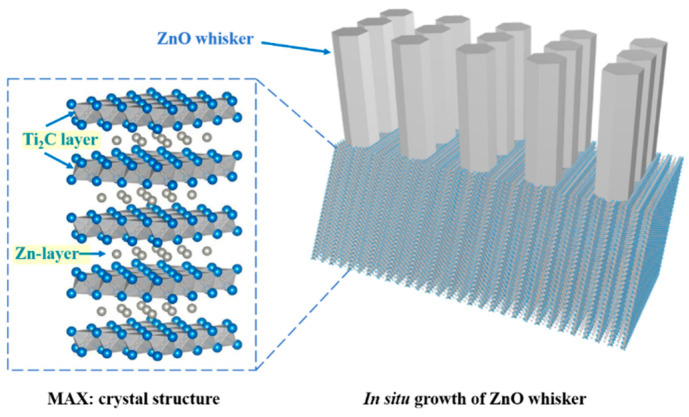
Illustrations of MAX phase crystal structure and the in-situ ZnO whisker growth.

Figure 1a shows the XRD pattern of the as-synthesized Ti_2_ZnC, which is consistent with the work of Huang et al. [37]. Figure 2a shows the SEM characterization of the as-synthesized Ti_2_ZnC. After being heated at 400 °C for 48 h, the Ti_2_ZnC sample was characterized again, and new XRD peaks indexed to ZnO appeared, as shown in Figure 1b. The SEM image of the sample after heating, shown in Figure 2b, shows that some Zn atoms diffuse out from the lattice points of Ti_2_ZnC and then are oxidized by oxygen in air, thus forming ZnO whiskers that are closely connected with the Ti_2_ZnC matrix. The ZnO whiskers grown on the Ti_2_ZnC correspond to the new XRD peaks of ZnO. Zhang et al. [40] reported that when Ti_2_SnC was heated to 400 °C, some Sn atoms would precipitate from the lattice points of Ti_2_SnC, and Ti_2_SnC was partially decomposed into Ti_2_C and Sn. Because the diffusion process mainly depends on temperature, the precipitation of Sn atoms is also strongly affected by temperature. The precipitation of Sn atoms is almost invisible under 200 °C. When the temperature rises to 400 °C, the precipitation of Sn atoms can be clearly observed. When the temperature rises to 600 °C, this phenomenon becomes more obvious. In this work, the soaking temperature was set to 400 °C to make Zn atoms diffuse out of the Ti_2_ZnC as much as possible. It is reasonable to ascribe this whisker growth process to the Zn atom precipitation from the lattice points of Ti_2_ZnC. Like other MAX phases, Ti_2_ZnC has a layered structure, as illustrated in Figure 3. The bonds between the Zn layer and Ti_2_C layer are relatively weak, which indicates that Ti-Zn bonds are easy to break under elevated temperature and some Zn atoms can diffuse out from the lattice points of Ti_2_ZnC. Then, the Zn atoms on the surface are oxidized by oxygen in the air, forming ZnO whiskers.

**Figure 4:**
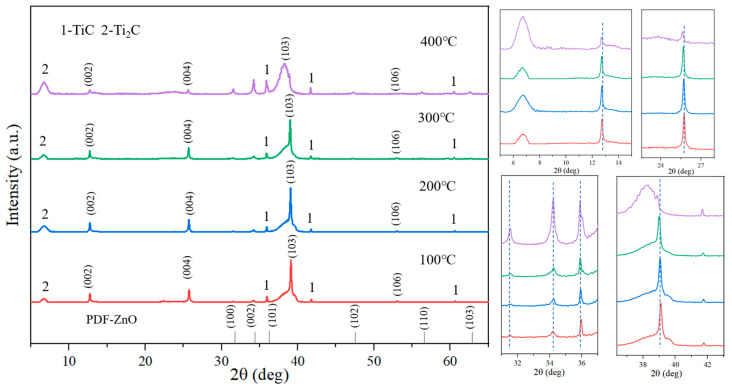
In-situ XRD pattern: Ti_2_ZnC heated at 100 °C, 200 °C, 300 °C and 400 °C, respectively.

Since the Zn atoms in the Ti_2_ZnC lattice are the Zn source for the growth of ZnO whiskers, how will the Ti_2_ZnC change with the increase in temperature during the heating process? The Ti_2_ZnC sample was characterized by in-situ XRD. The whole process was carried out from room temperature to 400 °C, the heating rate was 10 °C/min, and the sample was characterized at 100 °C, 200 °C, 300 °C and 400 °C, respectively. It can be seen from Figure 4 that the XRD results at four different heating temperatures all contain the diffraction peak of Ti_2_C (6.6°). The generation of this diffraction peak is due to the etching of the A-site atoms and the increase in the interlayer distance of the MAX phase. However, the existence of diffraction peaks corresponding to (002), (004) and (106) of Ti_2_ZnC at four temperatures means that only some Zn atoms diffuse out from the lattice points of Ti_2_ZnC, and Ti_2_ZnC and Ti_2_C exist in the sample at the same time. The partially enlarged figure on the right shows that with the increase in temperature, the diffraction peaks corresponding to (002), (004) and (103) of Ti_2_ZnC shift slightly to the left. Furthermore, with the increase in temperature, the intensity of the diffraction peaks of TiC and ZnO gradually increase, indicating that some Ti_2_ZnC decompose into TiC and Zn and Zn reacts with O_2_ in the air at high temperature, thus forming ZnO. This can be confirmed by the phenomenon that the intensity of the diffraction peaks corresponding to (100), (002), and (101) of ZnO increases with increasing temperature.

**Figure 5:**
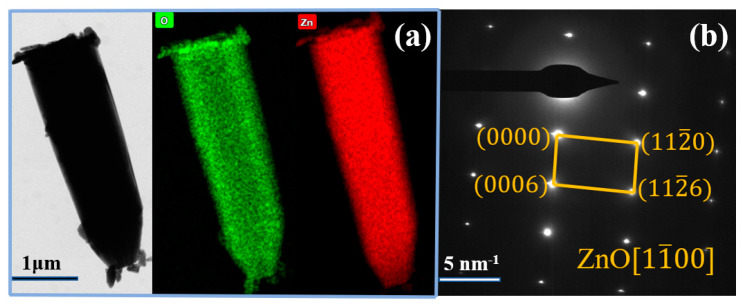
TEM characterization of (**a**) a ZnO whisker and its element mapping; (**b**) SAED pattern of the whisker.

Figure 5 shows a ZnO whisker observed under TEM. The corresponding element mapping (Figure 5b,c) shows that O and Zn elements are uniformly distributed throughout the whisker. The selected area electron diffraction (SAED) pattern of the whisker is shown in Figure 5b, which can be assigned to the ($11\bar{2}0$), (0006), and ($11\bar{2}6$) planes of ZnO along the [$1\bar{1}00$] axis, indicating that it is a single-crystal ZnO whisker.

**Figure 6:**
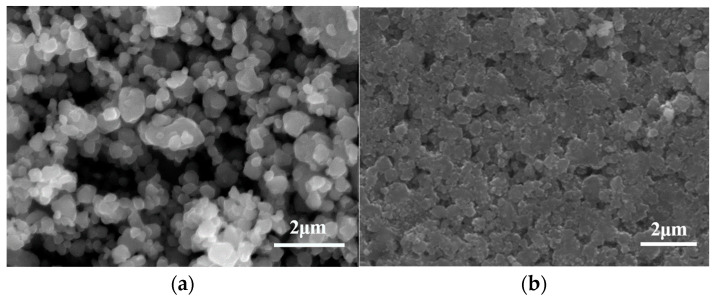
SEM characterization of (**a**) the ball-milled Ti_2_ZnC (18 h) and (**b**) the surface of the disc after cold-pressing.

Since the Zn atoms feeding the ZnO whisker growth are from the Ti_2_ZnC substrate, if we can make more Zn atoms come out of the substrate, more ZnO whiskers are expectable. Mechanically decomposing the MAX phase by ball-milling is an effective way to obtain small-sized carbides and A-site metal whiskers, and Tian et al. [25] reported that when Ti_2_SnC was ball-milled for 7 h, after cold-pressing at 1000 MPa and storing at room temperature (RT) for 30 days, a large number of Sn whiskers grew on the surface of the substrate. The study shows that with the increase in ball-milling time, the damage degree of ball-milling to Ti_2_SnC is intensified, and more active Sn atoms release from the lattice points of Ti_2_SnC, which serve as the source of Sn whisker growth. The same phenomenon also occurs in Ti_2_InC. Therefore, ball-milling was employed to mechanically decompose Ti_2_ZnC. The typical morphology of Ti_2_ZnC particles after ball-milling is shown in Figure 6a. Compared with Figure 2a, the size of Ti_2_ZnC particles is greatly reduced by ball-milling. Figure 1c shows the XRD of the ball-milled Ti_2_ZnC. Comparing the XRD data of Ti_2_ZnC before (Figure 1a) and after ball-milling (Figure 1c), it is found that the XRD peaks of the samples are broadened after ball-milling, indicating that the Ti_2_ZnC has been refined to a great extent. This result is consistent with the particle refinement observed in Figure 6a. In addition, the diffraction peaks of Zn can also be seen, indicating that the mechanochemical decomposition of Ti_2_ZnC has occurred and more Zn atoms are released from the lattice of the Ti_2_ZnC after mechanical exfoliation.

**Figure 7:**
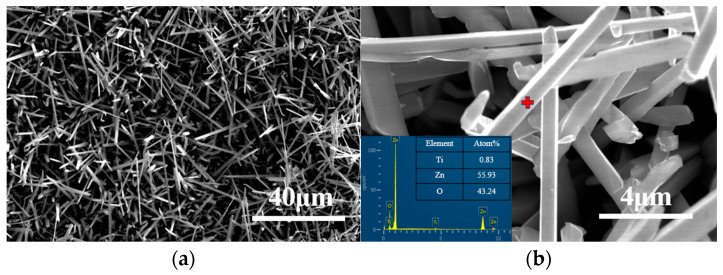
SEM characterization of (**a**) ZnO whiskers grown on the ball-milled (18 h) Ti_2_ZnC sample after dwelling at 400 °C for 48 h and (**b**) the grown ZnO whisker together with its EDS analysis result (the red plus sign).

Then, the ball-milled mixture was cold-pressed into discs under 800 MPa (Figure 6b) and heated at 400 °C for 48 h in the air. In Figure 7a, it can be seen that a large number of whiskers grow on the surface of the sample, and the EDS analysis result (Figure 7b) suggests that the whisker is composed of Zn and O elements, with Ti impurity accounting for 0.83 at.%. In addition, Figure 1d shows that the ZnO diffraction peaks are very obvious, indicating that these whiskers are ZnO whiskers. Because a large number of ZnO whiskers grown on the surface of the sample cover the Ti_2_ZnC matrix and Ti_2_ZnC has poor crystallinity after ball-milling while ZnO has high crystallinity, Figure 1d shows very strong ZnO diffraction peaks, while the diffraction peaks of Ti_2_ZnC are almost invisible. There are two reasons for the growth of a large number of ZnO whiskers. On the one hand, Ti_2_ZnC was mechanically decomposed and many more Zn atoms came out of the lattice of the Ti_2_ZnC after ball-milling, and these Zn atoms combine with O atoms to form whiskers. On the other hand, according to Figure 2b, some Zn atoms released from the lattice points of Ti_2_ZnC under 400 °C, and these Zn atoms contributed to the growth of ZnO whiskers. From Figure 7, it is found that the ZnO whiskers were longer and thinner than those observed in Figure 2b. This is because the reduced size of Ti_2_ZnC particles after ball-milling provides more sites for whisker growth.

**Figure 8:**
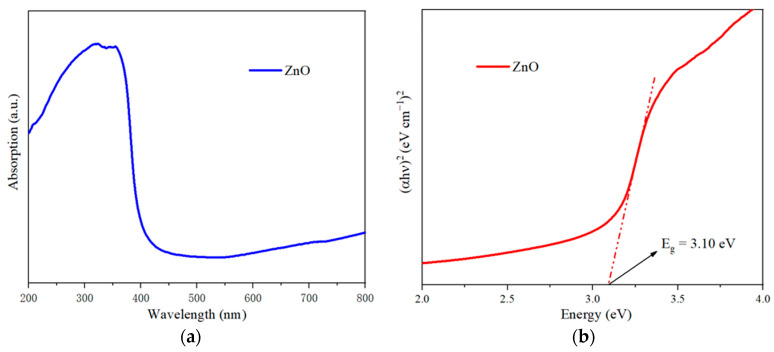
(**a**) UV-Vis spectra, and (**b**) Tauc plot of the grown ZnO whiskers.

The ZnO whiskers grown on the surface of the sample were collected to characterize their optical properties. The UV-Vis spectra for the grown ZnO whiskers are shown in Figure 8a, which shows that the grown ZnO whiskers possess very strong absorption for UV light. The band gap energy is calculated by Tauc’s plot, to obtain (αhν)^2^ vs hν graphs and extrapolate the linear segment of the curves. Figure 8b shows the Tauc’s plot of the grown ZnO whiskers, and the band gap of the grown ZnO whiskers is calculated to be 3.10 eV.

**Figure 9:**
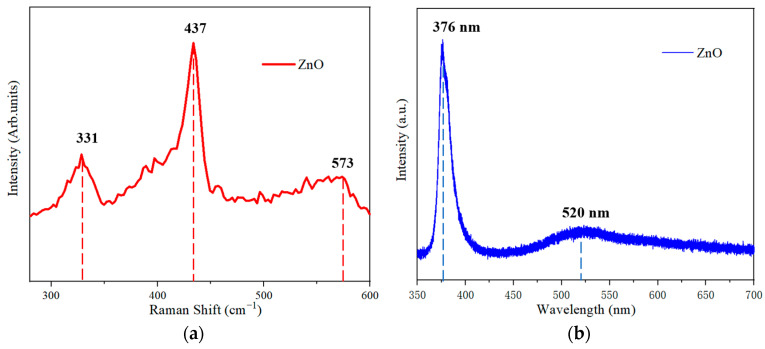
(**a**) Raman spectra, and (**b**) PL spectra of the grown ZnO whiskers.

The Raman spectra of the grown ZnO whiskers are displayed in Figure 9a. The whiskers exhibit a characteristic wurtzite ZnO Raman peak at 437 cm^−1^. In addition, the low-intensity peaks observed at 331 cm^−1^ and 573 cm^−1^ can be attributed to the presence of different types of defects such as oxygen vacancies and interstitials [41]. The photoluminescence spectroscopy (PL) spectra of the grown ZnO whiskers are shown in Figure 9b with λ_ex_ = 325 nm. The spectra are characterized by a strong emission peak centered around 376 nm, which is attributed to the UV near-band-edge emission of ZnO. In addition, there is a wide green emission peak around 520 nm, which can be attributed to the presence of defects [42]. The intensity of the green emission peak is lower than that of the UV emission peak, indicating that the ZnO whiskers grown on the surface of the sample have low defect concentrations and good crystallinity.

The ZnO whisker growth suggests the low-temperature instability of the Ti_2_ZnC, similar to Ti_2_SnC [40]. Specifically, when the grain size is reduced, more Zn atoms can diffuse out of the Ti_2_ZnC, and then oxidized, resulting in more ZnO whiskers. This consolidates the growth mechanism proposed for the formation of massive Sn whiskers found in Ti_2_SnC [24]. The findings here, on the one hand, may be harnessed to engineer a new method for the in-situ preparation of ZnO whiskers based on the Zn-containing MAX phase, and on the other hand, would help us to better understand the stability of Ti_2_ZnC and the whiskering mechanism of MAX phases.

## 4. Conclusions

In this study, the in-situ growth of ZnO whiskers on Ti_2_ZnC was realized by heating Ti_2_ZnC at 400 °C for 48 h. Ball-milling (700 rpm, 18 h) can reduce the size of Ti_2_ZnC particles and therefore extract more Zn atoms from the lattice points of Ti_2_ZnC, resulting in the growth of a large number of ZnO whiskers on the surface of Ti_2_ZnC. The ZnO whiskers grown on the surface of the sample (ball-milled and aged at 400 °C for 48 h) are thinner than those grown on the directly heated Ti_2_ZnC substrate (aging at 400 °C for 48 h), which is because the reduced size of Ti_2_ZnC particles after ball-milling provides more sites for whisker growth. This phenomenon could be harnessed to develop a new method for the in-situ preparation of ZnO whiskers. The findings here will also assist us in comprehensively understanding the stability of Ti_2_ZnC and promote the study of the whiskering mechanism of MAX phases. Additionally, the in-situ growth of ZnO on Ti_2_ZnC is expected to be promising for applications that require the immobilization of ZnO whiskers.

## Data Availability

Not applicable.
